# Mental distress and associated factors among college students in Kemisie district, Ethiopia

**DOI:** 10.1038/s41598-022-21710-6

**Published:** 2022-10-20

**Authors:** Aziza Siraji, Asressie Molla, Wolde Melese Ayele, Natnael Kebede

**Affiliations:** 1 Department of Reproductive Health, Kemisie General Hospital, Oromia Special Zone, Kemise, Ethiopia; 2 Department of Epidemiology and Biostatistics, College of Medicine and Health Sciences, Wollo University, Dessie, Ethiopia; 3 Department of Public Health, College of Medicine and Health Sciences, Injibara University, Injibara, Ethiopia; 4 Department of Health Promotion, School of Public Health College of Medicine Health Sciences, Wollo University, Dessie, Ethiopia

**Keywords:** Health care, Medical research

## Abstract

Mental health problems such as distress affect society in a non-differential fashion. In recent decades, mental distress is becoming a common health problem among students. In this regard, there is limited information about the problem available in Ethiopia. Therefore, this study aimed to determine the prevalence and associated factors of mental distress among college students in Kemisie district, Ethiopia. An institution-based cross-sectional study was conducted among 408 students from February 11 to 14 2020. A stratified sampling technique was used to select the study participants. Data were collected using a structured self-administered questionnaire. Self-reporting questionnaire (referred to as the SRQ-20) is a standardized questionnaire having 20-item questions and was used as a tool for mental distress. Appropriate descriptive statistics was done. A binary logistic regression model was used to identify factors associated with mental distress. An adjusted odds ratio with a 95% confidence interval was computed to determine the level of significance. A *p* value < 0.05 was used to declare the statistical significance of the variables. Prevalence of mental distress among students was 17.6% (95% CI 13.8–21.4%). Not having close friends (AOR = 3.61; 95% CI 1.61–8.14), attend religious programs (AOR = 0.23; 95% CI 0.14–0.53), conflict with friend (AOR = 3.07; 95% CI 1.44–6.33), not having pocket money (AOR = 2.72; 95% CI 1.27–25.80), ever use of Chat (AOR = 5.06; 95% CI 2.12–11.80), current use of Chat (AOR = 3.12; 95% CI 1.04–9.82), decreased grade than anticipated (AOR = 3.20; 95% CI 1.436–7.16), and low and moderate social support (AOR = 3.34; 95% CI 1.41–7.92) and (AOR = 1.47; 95% CI 1.08–5.68), respectively were statistically significantly associated factors of mental distress. The overall prevalence of mental distress among students in Kemisie district, Ethiopia was high. In Ethiopia, along with the current economic crisis and the absence of social support, the problem could be increased. Therefore, the mental health needs of the college students require attention with special emphasis on not having close friends, never attending religious programs, conflict with friends, absence of pocket money, students who use Khat, and those who have low social support.

## Introduction

Mental health is defined as "the successful performance of mental functions related to thinking, emotions, and behaviors that lead to productive activities, good relationships with others, and the ability to adapt, change, and transform coping with adversity”^1^. Mental distress is one of the mental health problems that affect society non-differentially. No vaccine boosts immunity to prevent mental health disorders. The problem of mental distress is higher among the poor, homeless, the unemployed, people with low education, victims of violence and adolescents, and abused females^2^. Mental distress is a common phenomenon among college students. Empirical research confirms this the college student population has a higher prevalence of mental disorders than the general population^3^.

Mental illness can have far-reaching effects function of college students. On a personal level, they can affect all aspects of physical, emotional, cognitive, and interpersonal interactions. You can also harm school performance^4^.

Currently, mental distress is an important public health problem. Globally, more than 450 million people have suffered from mental distress in the past decades. At present, it is a leading cause of disability worldwide, accounting for more than 30% of disabilities^5^. Mental distress affects a large portion of the world's population and is prevalent among college students around the world^6^.

According to the findings from the previous studies, the trend of mental distress is increasing over time. A study on the American population indicated that extreme distress rose from 3.6% in 1993 to 6.4% in 2019^7^.

In Africa, mental illness is an under-recognized public health challenge. Studies conducted in South Africa revealed that the prevalence of common mental disorders is 27% among students^6^. Recently, the number of students with psychological problems on campus has increased^8^. The studies revealed that 41.9% of students in Malaysia^9^, 25.7% of students in France^10^, 17.1% in Egypt^11^, 16.2% in Uganda^12^, and 10.8% in Kenya^13^ of the students experienced mental distress.

In Ethiopia, however, mental health is one of the least favorable health programs in higher education institutions, although more than one-third of college students have been affected by a mental health problem at least once in their campus life, whether institutional or trained^14^. Likewise, in Ethiopia, higher academic institution studies revealed the prevalence of mental distress among students with a wide variation of 21.6% in Adama^15^, 29% in Mizan Aman^5^, and 40.9% in Gondar^6^.

According to the findings of previous research, socio-demographic^16,17^, behavioral^8,10^, academic-related^18,19^, and social support^20,21^, factors are the prominent factors for mental distress.

At present, mental health service is incorporated into the national health policy of Ethiopia. However, interventions are too limited, and the lack of evidence-based information about the problem is a contributory factor to lower mental health services^5^. Accordingly, scientifically explored data that showed the magnitude of mental health problems among students is required in Ethiopia. Therefore, this study aimed to assess the prevalence and associated factors of mental distress among College students in Ethiopia.

The study finding will have credit for getting reliable data about the burden of mental distress among students that will guide interventions to decrease the risk. The intervention in turn will improve students' psychological well-being. Moreover, the data will be used as a source to search for counteractive action from policymakers, college officials, non-governmental organizations, parents, students, and other concerned bodies such as religious institutions.

## Methods

### Study design and setting

An institution-based cross-sectional study was carried out among college students in Kemisie from February 11 to 14, 2020. Kemisie is located 290 km northeast of Addis Ababa, the capital city of Ethiopia. Kemisie College of teacher education was established in 2011. The college is delivering educational services for more than 600 regular and 500 extension students (extension: students who learn at the weekend).

### Study population and instrument

The source populations were all students attending Kemisie College of teacher education. Those students who left the college due to withdrawal or academic dismissal were excluded from the study. Data were collected using a structured self-administered questionnaire having five parts (Additional file 1). The first part contains the socio-demographic characteristics of students. The second part of the tool was a Self-Reporting Questionnaire, which was used to assess the prevalence of mental distress among students. This self-reporting questionnaire (referred to as the SRQ-20) is a standardized questionnaire having 20-item questions, originally developed by WHO to indicate mental distress^22^. Those students who were found to have 8 or more symptoms of the 20 items in the last four weeks were considered to have mental distress. This cut**-**off point was used based on the reports from the SRQ-20 adoption study on Ethiopian populations^23^. The third part of the questionnaire was about the student's behavior. The fourth part of the questionnaire assessed their academic factors. The last part was designed to assess social support using a validated 12-item Multidimensional Scale of Perceived Social Support Tool^24^. This part is divided into factor groups relating to the source of social support such as family, friends, and significant others. Each item was scored from Very strongly disagree (1), Strongly disagree (2), Mildly disagree (3), Neutral (4), Mildly agree (5), strongly agree (6), and Very strongly agree (7) Likert scales. The scales were then changed to low, moderate, and high social support. Participants who scored 12–48, 49–68, and 69- 84 were considered as having low, moderate, and high social support, respectively^24^. The detailed operational definitions of terms are presented.

First, the tool was prepared in English and then translated into two local (Amharic and Oromia) languages. Copies of the three versions were used for data collection. The student's language preference was assured to collect the data and maintain the quality of data.

### Sample size determination and sampling procedure

The sample size was determined by using a single population proportion formula considering the following assumptions: 95% confidence level, 5% tolerable margin of error, and 40.9% prevalence of mental distress among undergraduate students of the University of Gondar^6^. The sample size was also attempted to determine using the second objective. However, the calculated sample sizes were less than the sample size obtained by the first objective. After adding a 10% non-response rate, the final sample size was 408.

A stratified sampling technique was used to classify students based on their department. The stratification was based on the assumption of a difference in mental distress across the departments. Accordingly, the students were classified into five strata (i.e. sports science, natural science, mathematics, professional, and language). The sample was allocated to each stratum based on proportional to the population size in each department. A simple random sampling technique was to select the study subjects in each department. The list of active students was obtained from the respective department (Additional file 2).

#### Operational definition

*Mental distress* In this study, students who were found to have 8 or more symptoms of the 20 items self-reporting questionnaires (SRQ-20) in the last 4 weeks are considered as having mental distress. The cut point was used based on the reports from the validation study of SRQ-20. The tool was validated and used in the country^23^.

#### Substance use

- *Current users* when subjects use a specified substance at least once in the last 30 days.
- *Ever users* when students use a specified substance even once in their lifetime.
- *Social support* was measured using a 12-item Multidimensional Scale of Perceived Social Support^24^.
- *High level of social support* when subjects score 69–84 on the sum of social support scale.
- *Moderate social support* when subjects score 49–68 on the sum of the social support scale.
- *Low level of social support* when subjects score 12–48 the sum of the social support scale is divided into factor groups relating to the source of social support namely family, friends, and significant others. Each item is scored from one (Very strongly disagree) to 7 (very strongly agree). The total sum of all the 12 items possibly ranges from 12 to 84.
- *Religious practice* students who are involved in religious programs irrespective of their religion and frequency of practice.

### Data processing and analysis

Data were checked, coded, and entered using EPI data version 3.5.4, then transferred to SPSS version 24 for advanced analysis. Descriptive statistics like percentage, mean, and standard deviation were computed and presented by tables and figures. This study had two outcomes. The first was mental distress, while the second had four subsections which were socio-demographic characteristics, behavioral, academic, and social independent factors. Binary logistic regression was performed to see the association between the independent variables and the dependent variable. A multivariable logistic regression analysis was performed after the variable showed a *p* value of ≤ 0.2 in the bivariable analysis to control the confounders and identify the independent factors. *p* Value less than 0.05 and adjusted odds ratios (AOR) with a 95% confidence interval (CI) were used to declare the presence of statistical significance.

### Ethical approval and consent to participate

Ethical clearance was insured from Wollo University, College of Medicine and Health Sciences ethical review committee with the reference number of Ref no: CMHS/85/13/2020. The ethical review committee confirmed that verbal consent could be used unless personal identifiers are used. Similarly, a written permission letter was obtained from Kemisie College of teacher education. Informed consent was obtained from all study participants. However, we have not used personal identification numbers (ID) and names during data collection for personal anonymity. All over; the risks, burdens, and benefits that the participants will take were following the Declaration of Helsinki. The collected data have been kept confidential.

## Results

### Socio-demographic characteristics of the respondents

Three hundred and ninety-eight respondents participated in the study, making a response rate of 97.54%. The mean (± SD) age of the respondents was 20 + 1.54 years. About 330 (82.9%) of the students were from rural settings. The majority, 159 (39.9%) of the participants were Islamic religion followers, and 331 (83.1%) took part in religious practice. Among the participants, 176 (44.22%) and 242 (60.8%) were from the natural science department and the second year of academic enrollment, respectively. Moreover, 131 (32.9%) of the respondents had no boy or girlfriend. Two hundred ninety-four (73.9%) of the respondents had pocket money ranging from a minimum of 350 to a maximum of 700 Ethiopian Birr per month. Roughly, 165 (41.4%) of the respondents had a family history of mental illness (Table 1).

**Table1:** Socio-demographic characteristics of the respondents (n = 398).

Variables	Frequency	Percent (%)
Age in years, Mean ± SD, (Range)	20.5 ± 1.2, (18–24)	
18–20	198	49.7
21–25	200	50.3
**Sex**		
Male	198	49.7
Female	200	50.3
**Residency**		
Urban	68	17.1
Rural	330	82.9
**Religion**		
Orthodox	126	31.7
Muslim	159	39.9
Protestant*	113	28.4
**Department**		
Natural science	70	17.6
Language	176	44.2
Sport science	69	17.3
Mathematics	51	12.8
Professional	32	8.1
**Year of enrolment**		
Second	242	67.1
Third	156	32.9
**Have a boy/girlfriend**		
Yes	331	83.2
No	67	16.8
**Have close friends**		
Yes	245	61.6
No	153	38.4
**Have religious practice**		
Yes	331	83.2
No	67	16.8
**Number of students per class**		
< 50	85	21.4
50–80	313	78.6
**Ever had a conflict with friends?**		
Yes	184	46.2
No	214	53.8
**Have pocket money?**		
Yes	294	73.9
No	104	26.1
**Amount of money per Month in Ethiopian Birr (n = 294)**		
Mean ± SD, Range	502.7 ± 85.5, (50–700)	
50–399	69	23.5
400–700	225	76.5
**Have financial distress**		
Yes	155	38.9
No	243	61.1
**Have a family history of mental health problems**		
Yes	165	41.5
No	233	58.5

*One Catholic follower is included.

### Prevalence of mental distress

**Figure 1:**
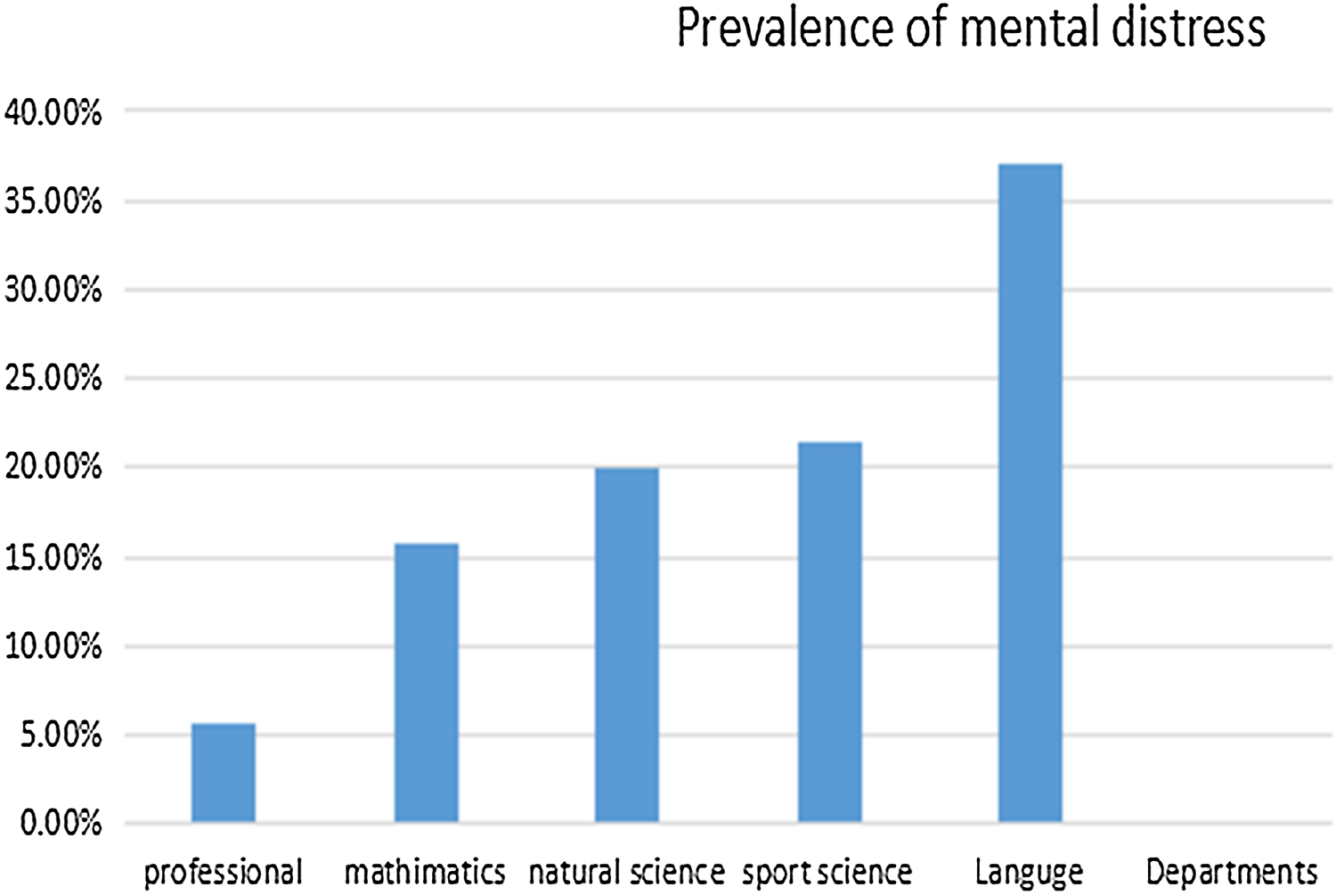
Prevalence of mental distress among college students in Kemisie district across department.

The prevalence of mental distress among students was 17.6% (95% CI 13.8–21.4%). A high prevalence of mental distress was reported in the language department 147 (37.1%). As shown in Fig. 1, the lowest prevalence of mental distress was found among the participants from the professional department, 20 (5.7%). Roughly, a higher proportion of mental distress was among females than males (18.8% vs. 16.3%) (Fig. 1).

### Factors associated with mental distress

In the bivariable logistic regression analysis, year of enrollment, having a boy or girlfriend, having close friends, religious practice, ever had a conflict with friends, absence of pocket money, financial distress, ever use of chat, current use of chat, decrease grade than anticipated, increase class workload, a serious argument with the instructor, missed too many class, and social support were the factors associated with mental distress.

However, in multivariable analysis, close friends, religious practice, conflict, pocket money, current and ever use of chat, decrease grades than anticipated, and social support remained as statistically significantly associated factors of mental distress among respondents (Table 2).

**Table 2:** Bivariable and multivariable logistic regression analysis of factors associated with mental distress; (n = 398).

Variables	Mental distress		COR (95% CI)	AOR (95% CI)	*p* Value
	Yes	No			
**Have a boy or girlfriend?**					
Yes	53	214	1	1	
No	17	114	0.60(0.33–1.08)	0.58(0.14–1.54)	0.07
**Have close friends**					
Yes	34	211	1		
No	36	117	1.97(1.13–3.21)	3.61(1.61–8.14)*	0.002
**Religious practice**					
Yes	53	218	0.15(0.09–0.25)	0.23(0.14–0.53)*	0.002
No	77	50	1	1	
**Ever had conflict**					
Yes	47	137	2.85(1.65–4.91)	3.07(1.44–6.33)*	0.001
No	23	191	1	1	
**Have pocket money**					
Yes	45	249	1		
No	25	79	1.75(1.01–3.03)	2.72(1.27–5.80)*	0.001
**Financial distress**					
Yes	34	121	1.61(0.96–2.71)	1.12(0.57–2.19)	
No	36	207	1	1	0.73
**Ever use of chat**					
Yes	24	49	2.97(1.66–5.31)	5.06(2.12–11.80)*	0.001
No	46	279	1	1	
**Current use of chat**					
Yes	13	18	3.92(1.82–8.46)	3.12(1.04–9.82)*	0.04
No	57	310	1	1	
**Increased class workload**					
Yes	19	160	0.45(0.22–0.69)	0.53(0.25–1.11)	0.09
No	51	168	1	1	
**Decreased grade than antic**					
Yes	50	159	2.65(1.51–4.66)	3.20(1.436–7.16)*	0.04
No	20	169	1	1	
**Missed many classes**					
Yes	22	178	0.38(0.22–0.66)	0.38(0.62–1.09)	0.42
No	48	151	1	1	
**Serious argument with instructor**					
Yes	23	50	2.53(1.41–4.51)	3.11(1.73–6.61)*	0.001
No	50	275	1	1	
**Social support**					
Low	39	83	4.89(2.42–9.89)	3.34(1.41–7.92)*	0.01
Moderate	19	120	1.64(0.77–3.09)	1.47(1.08–5.68)*	
High	12	125	1	1	

*Statistically significant at *p* < 0.05.

## Discussion

Mental distress is an important public health problem and is becoming a common health problem among college students. In the current study, the prevalence of mental distress among students was found to be 17.6% (95% CI 13.8–21.4%). This result is similar to the study in Egypt 17.1%^11^, and Adama, central Ethiopia (21.6%)^15^. However, this finding is lower compared to studies in Malaysia (41.9%)^9^ USA (57%)^25^, and Australia (53%)^26^. This difference can be attributed to the different socio-cultural and economic development of countries. This result was also lower compared to studies in southern Ethiopia (29.2%)^5^ and 30%^27^, and Northern 40.9%^6^ Ethiopia regions. This disagreement might be due to the improvement of infrastructure and a service option provided by school authorities from time to time. Similarly, the former studies were conducted at the university level, in which students could decrease much stress by invoking thoughts such as food, reading materials, and social interactions.

In contradiction, the prevalence of mental distress in the current study is higher than the finding from studies in Vietnam (10.7%)^28^ and Kenya 10.8%^13^. This could be due to the different tools used to collect the data and the variations in the study period. In this particular study; not having close friends, never attending religious programs, conflict with friends, absence of pocket money, ever and current use of Chat, a lower grade than anticipated, and low and moderate social support were important predictors of mental distress.

This study found that mental distress was strongly associated with having close friends. The students who have no close friends were 3.6 times more than three times more at risk of developing mental distress than those who had close friends. This finding is supported by previous studies in Mizan Tip and Adama^5,15^, the current study could be because participants who had no close friends were more at risk of mental distress.

In this study, religious practice was an independent predictor of mental distress. Students who were involved in the religious program were 77% less likely to be mentally distressed. This result is congruent with the studies done in Ethiopia^15,27^ which identified that students who were involved in religious practice were less likely to have mental distress. This issue is intermingled because religious teaching and bits of advice help in stress management and as well as could smooth the development of adaptive behaviors^9^, resulting in less or no mental distress.

This study revealed that the likelihood of mental distress was 3 times higher among students who had a conflict with their friends. This result was built on what was noted by studies in Adama University, Ethiopia^7^ and the University of Gondar, Ethiopia^6^. Peer conflict might increase stress. The students might worry about how they resolve the conflict, or they might fear the consequence of the conflict. Moreover, this study was conducted in a situation in which political instabilities are observed in Ethiopia that resulted in conflicts among students. This will lead to mental distress.

Although it is not simple to give a sound explanation, an important result in this research was that having no pocket money was an independent predictor of mental distress. Those students who had no pocket money were 2.72 times more likely to experience mental distress. This finding is supported by studies in Australia^29^, the USA^30^, Nigeria^31^, and Ethiopia^6^. The rising cost of learning materials, recreation services, and the student's belief that they are helpless may create a stressful situation for students. This justification is more supported by a study^31^ that students who have no pocket money experience anxiety, frustration, and a sense of haplessness and disturbance in sleeping which may further lead students to be mentally distressed.

Moreover, the odds of mental distress were higher among Chat chewer students than non-chewers. Students who ever use chat and currently use chat were 5.06 and 3.12 times more likely to have mental distress as compared to their counterparts, respectively. This finding is in line with the previous studies in Ethiopia^7,32^. Also, the finding in Norwegian is built on the results^33^, where Chat chewing was a significant predictor of mental distress. This may be because substance such as Chat use leads to inefficiency in life career, impaired relationship, and sleep difficulty.

Furthermore, the academic features of the students were found to be the other factors associated with mental distress. Students whose grade was lower than anticipated were three times more at risk to experience mental distress than their counterparts. The result is consistent with a study finding in the University of Gondar^6^.

Though the current study does not show any significant association between class overload and mental distress, a study at the University of Gondar^6^ identified that class overload was statistically a cause of mental distress.

Finally, this study identified that social support was another determinant for mental distress among college students. Students with low and moderate social support were more likely to be at risk for mental distress as compared to those students who had high social support. The finding is congruent with the findings in the studies^34,35^. According to the result of a study in Norwegian^23^, social support is important for maintaining good physical and mental health. Besides, social support may moderate genetic and environmental vulnerabilities and confer resilience to stress.

In contradiction to the studies^6,33^, the current study identified that family history of mental distress was not statistically significantly associated with mental distress among the students. This discordancy might be due to the socioeconomic difference of the population and the study period variation.

In addition, unlike the finding from the studies^7,36^, this study found that the likelihood of mental distress was higher among students who had a serious argument with their teachers. This disagreement might be due to the difference in sample size, socioeconomic factors, and study years. However, this result is similar to a study on Aksum University students^19^.

## Limitation of study

The investigators of this study noted that readers should consider the following possible limitations. First, since a cross-sectional study design was used, it is difficult to ascertain the temporality problem. Second, some replies were depending on the respondents' remembrance which might be prone to recall bias. Finally, social desirability bias due to some sensitive variables such as disclosing Chat chewing might be introduced.

## Conclusions

The overall prevalence of mental distress among students in Kemisie district, Ethiopia was high. Not having close friends, never attending religious programs, conflict with friends, absence of pocket money, ever and current use of Chat, a lower grade than anticipated, and low and moderate social support were factors statistically associated with mental distress. Therefore, designing prevention and treatment programs tailored to different circumstances and due attention is essential. The mental health needs of the college students require attention with special emphasis on not having close friends, never attending religious programs, conflict with friends, absence of pocket money, students who use Khat, and those who have low social support.

## Supplementary Information

Supplementary Information.

## Data Availability

All the necessary data are included in the manuscript. The raw data are accessible to the corresponding author with a reasonable request.

## Abbreviations

AOR: Adjusted odds ratio
CI: Confidence interval
COR: Crude odds ratio
SRQ: Self reporting questions
