# Supplementary material for: Mental distress and associated factors among college students in Kemisie district, Ethiopia
Source: Sci Rep. 2022 Oct 20;12:17541. doi: 10.1038/s41598-022-21710-6 (PMC9585086; doi:10.1038/s41598-022-21710-6)
Supplement: Supplementary file 1 — Supplementary Information. [file 41598_2022_21710_MOESM1_ESM.doc]

**Additional file 1. The English version Questionnaire**

**Part 1.** Socio-demographic characteristics of the respondents

This part of the questionnaire assesses about your socio economic and demographic information’s, after reading the following the questions please give appropriate answer concerning your socio economic and demographic information.

| **No** | **Questions** | **Response/Options** | **Remark** |
| --- | --- | --- | --- |
| 101 | Age in years | -------------- |  |
| 102 | sex | 1. Male  2. Female |  |
| 103 | Residence | 1. Urban  2. Rural |  |
| 104 | Religion | 1. Orthodox  2. Muslim  3. Protestant  4. Catholic  5. Other_______ |  |
| 105 | From which department you? | 1. natural science  2. language  3. sport science  4 mathematics |  |
| 107 | Year of enrollment | 1. 2nd year  2. 3rd year |  |
| 108 | Are you interested with your  department | 1. Yes  2. No |  |
| 109 | Do you have boy or girl friend | 1. Yes  2. No |  |
| 110 | Do you have close friends | 1. Yes  2. No |  |
| 111 | Do you take part in religious  practice | 1. Always  2. Often  3. Some times  4. Never |  |
| 112 | How many students are there in  your class room? | 1. <50  2. 50-80  3. >80 |  |
| 113 | Do you face conflict with your  friends in the class  rooms | 1. Always  2. Often  3. Some times  4. Never |  |
| 114 | Do you have pocket money? | 1. Yes  2. No |  |
| 115 | If yes for question no 115, what is  your monthly amount of pocket  money in birr | __________ |  |
| 116 | Do you have financial  distress/problem for stationary (like  photocopy) and recreational  activity | 1. Yes  2. No |  |
| 117 | Is there any family history of  mental illness | 1. Yes  2. No |  |

**Part 2:** Self-Reporting Questionnaire (SRQ)

The following questions are related to certain pains and problems that may have bothered you in the last 30 days. Please tick the ‘Yes’ box if you have had this symptom in the last 30 days. On the other hand, if you have not experienced this symptom in the last 30 days please tick the ‘No’ box.

| **No** | Encountered health Problem with in the last 4 weeks | yes | No |
| --- | --- | --- | --- |
| 201 | Do you often have head ache? |  |  |
| 202 | Is your appetite poor? |  |  |
| 203 | Do you sleep badly? |  |  |
| 204 | Are you easily frightened? |  |  |
| 205 | Do your hands shake? |  |  |
| 206 | Do you feel nervous, tens or worried? |  |  |
| 207 | Is your digestion poor? |  |  |
| 208 | Do you have trouble thinking clearly? |  |  |
| 209 | Do you unhappy? |  |  |
| 210 | Do you cry more than usual? |  |  |
| 211 | Do you find it difficult to enjoy your daily activities? |  |  |
| 212 | Do you find difficult in decision making in day to day life? |  |  |
| 213 | Is your daily work suffering? |  |  |
| 214 | Are you unable to play a useful part in life? |  |  |
| 215 | Have you lost interest in things? |  |  |
| 216 | Do you feel that you are a worthless person? |  |  |
| 217 | Has the thought of ending your life been on your mind? |  |  |
| 218 | Do you feel tired all the time? |  |  |
| 219 | Do you have uncomfortable feelings in your stomach? |  |  |
| 220 | Are you easily tired |  |  |

**PART 3:** Behavioral factor

The following questions focuses on Chat chewing practices, Alcohol drinking, Cigarette smoking and other substances like Hashish. So you are kindly requested to give a real answer about your personal behavior on the use of these substances.

| no | Please tick the ‘Yes’ box if you have had use and ‘No’ box if you don’t use the mentioned substance in your life time or in the last 1 month as directed. | yes | no |
| --- | --- | --- | --- |
| 301 | Have you ever used khat in your life? |  |  |
| 302 | Have you used Khat in the last 1 month? |  |  |
| 303 | Have you ever used alcohol drinks (like Arekie, TelaTejbeer, or any other alcohol drinks) in your life time |  |  |
| 304 | Have you used any kind of alcohol drinks in the last 1month? |  |  |
| 305 | Have you ever used Tobacco products such as cigarette smoking, shisha |  |  |
| 306 | Have you used any kind of tobacco product in the last 1month? |  |  |
| 307 | Have you ever used other substances / Such as hashish in your life time |  |  |
| 308 | Have you used any other substances / Such as hashish, in the last 1 month? |  |  |

**Part 4.** Academic factors

This part assesses about academic factors that students face/experience in the academic year. For the first six items (401-405) the answer is provided by “Yes” or “No”. For the last question (406) please write your last semester (if your academic year is semester based) or last year cumulative grade on the space provided.

| no | Please tick the **‘Yes’** box if you have experienced and **‘No’** box if you don’t in the last academic year. | yes | no |
| --- | --- | --- | --- |
| 401 | Increase class work load |  |  |
| 402 | Decrease grade than anticipated |  |  |
| 403 | Missed too many class |  |  |
| 404 | Serious arguments with instructors |  |  |
| 405 | Lack of vacations /break |  |  |
| 406 | What is your last semester/ year cumulative grade(CGPA) | ------------ | |

**Part 5:** Social support

This part assesses about social support that the respondents have got from their family, friends, and significant others. Each item is scored from 1(very strongly disagree to 7 (very strongly agree). Read each statement carefully and indicate how you feel about each stamen.

| no | Very strongly disagree (1), Strongly disagree(2), Mildly disagree (3), Neutral(4),Mildly agree (5), strongly agree (6), Very strongly agree (7) | | | | | | | | | |
| --- | --- | --- | --- | --- | --- | --- | --- | --- | --- | --- |
| 501 | There is a special person who is around whenI am in need | 1 | 2 | 3 | | 4 | 5 | 6 | | 7 |
| 502 | There is a special person with whom I can share my joys and sorrows | 1 | 2 | 3 | | 4 | 5 | 6 | | 7 |
| 503 | My family really tries to helpme | 1 | 2 | 3 | | 4 | 5 | 6 | | 7 |
| 504 | I get emotional help andsupport I need frommy family | 1 | 2 | 3 | | 4 | 5 | 6 | | 7 |
| 505 | I have a special person who is a real sourceof comfort to me | 1 | 2 | 3 | | 4 | 5 | 6 | | 7 |
| 506 | My friends really try to help me | 1 | 2 | 3 | | 4 | 5 | 6 | | 7 |
| 507 | I can count on my friends when things gowrong | 1 | 2 | 3 | | 4 | 5 | 6 | | 7 |
| 508 | I can talk about my problems with my family | 1 | 2 | 3 | | 4 | 5 | 6 | | 7 |
| 509 | I have friends with whom I can share my joysand sorrows | 1 | 2 | 3 | | 4 | 5 | | 6 | 7 |
| 510 | There is a special person in my life who caresabout my feelings | 1 | 2 | 3 | | 4 | 5 | | 6 | 7 |
| 511 | My family is willing to help me makedecisions | 1 | 2 | | 3 | 4 | 5 | | 6 | 7 |
| 512 | I can talk about my problems with my friends | 1 | 2 | | 3 | 4 | 5 | | 6 | 7 |

*Note: DSC; Department of sport Science, DP; Department of Professional, DNS; Department of Natural Science, DM; Department of Mathematics, DL: Department of Language, SRS: simple Random Sampling*

**Additional file 2. Sampling procedure**

DSC=115

DP (KG)=59

0

-

0

DNS=114

DM=82

DL=293

**KCTE=663**

71

36

70

51

180

Stratification by Department

Total number of department with total number of students

Proportional to sample size allocation

Study subjects selected by SRS

Figure 1: Schematic presentation of the sampling procedure to select the study participant
